# Dietary carbohydrate intake and mortality: a prospective cohort study and meta-analysis

**DOI:** 10.1016/S2468-2667(18)30135-X

**Published:** 2018-08-17

**Authors:** Sara B Seidelmann, Brian Claggett, Susan Cheng, Mir Henglin, Amil Shah, Lyn M Steffen, Aaron R Folsom, Eric B Rimm, Walter C Willett, Scott D Solomon

**Affiliations:** Cardiovascular Division, (S B Seidelmann MD, B Claggett PhD, S Cheng MD, M Henglin BA, A Shah MD, S D Solomon MD) and Channing Division of Network Medicine (E B Rimm ScD, W C Willett MD), Department of Medicine, Brigham and Women’s Hospital, Boston, MA, USA; Division of Epidemiology and Community Health, University of Minnesota, Minneapolis, MN, USA (L M Steffen PhD, A R Folsom MD); and Department of Epidemiology and Department of Nutrition, Harvard T.H. Chan School of Public Health, Boston, MA, USA (E B Rimm, W C Willett)

## Abstract

**Background:**

Low carbohydrate diets, which restrict carbohydrate in favour of increased protein or fat intake, or both, are a popular weight-loss strategy. However, the long-term effect of carbohydrate restriction on mortality is controversial and could depend on whether dietary carbohydrate is replaced by plant-based or animal-based fat and protein. We aimed to investigate the association between carbohydrate intake and mortality.

**Methods:**

We studied 15 428 adults aged 45–64 years, in four US communities, who completed a dietary questionnaire at enrolment in the Atherosclerosis Risk in Communities (ARIC) study (between 1987 and 1989), and who did not report extreme caloric intake (<600 kcal or >4200 kcal per day for men and <500 kcal or >3600 kcal per day for women). The primary outcome was all-cause mortality. We investigated the association between the percentage of energy from carbohydrate intake and all-cause mortality, accounting for possible non-linear relationships in this cohort. We further examined this association, combining ARIC data with data for carbohydrate intake reported from seven multinational prospective studies in a meta-analysis. Finally, we assessed whether the substitution of animal or plant sources of fat and protein for carbohydrate affected mortality.

**Findings:**

During a median follow-up of 25 years there were 6283 deaths in the ARIC cohort, and there were 40 181 deaths across all cohort studies. In the ARIC cohort, after multivariable adjustment, there was a U-shaped association between the percentage of energy consumed from carbohydrate (mean 48·9%, SD 9·4) and mortality: a percentage of 50–55% energy from carbohydrate was associated with the lowest risk of mortality. In the meta-analysis of all cohorts (432 179 participants), both low carbohydrate consumption (<40%) and high carbohydrate consumption (>70%) conferred greater mortality risk than did moderate intake, which was consistent with a U-shaped association (pooled hazard ratio 1·20, 95% CI 1·09–1·32 for low carbohydrate consumption; 1·23, 1·11–1·36 for high carbohydrate consumption). However, results varied by the source of macronutrients: mortality increased when carbohydrates were exchanged for animal-derived fat or protein (1·18, 1·08–1·29) and mortality decreased when the substitutions were plant-based (0·82, 0·78–0·87).

**Interpretation:**

Both high and low percentages of carbohydrate diets were associated with increased mortality, with minimal risk observed at 50–55% carbohydrate intake. Low carbohydrate dietary patterns favouring animal-derived protein and fat sources, from sources such as lamb, beef, pork, and chicken, were associated with higher mortality, whereas those that favoured plant-derived protein and fat intake, from sources such as vegetables, nuts, peanut butter, and whole-grain breads, were associated with lower mortality, suggesting that the source of food notably modifies the association between carbohydrate intake and mortality.

## Introduction

Some dietary guidelines have focused on lowering saturated and trans fat but not total fat or overall macronutrient composition.^1,2^ Other guidelines continue to recommend lowering total fat (<30% of energy from fat) in exchange for higher carbohydrate intake.^3^ In practice, however, low carbohydrate diets that exchange carbohydrates for a greater intake of protein or fat have gained substantial popularity because of their ability to induce short-term weight loss,^4–7^ despite incomplete and conflicting data regarding their long-term effects on health outcomes.^8–12^ Results from meta-analyses that included several large cohort studies in North America and Europe have suggested an association between increased mortality and low carbohydrate intake.^8–12^ However, the 2017 Prospective Urban Rural Epidemiology (PURE) study, of individuals from 18 countries across five continents (n=135 335, median follow up 7·4 years, 5796 deaths), reported that high carbohydrate intake was associated with increased risk of mortality.^13^ These data were interpreted as being contrary to previous work in the field, prompting calls for revisions to current nutrition guidelines.^13,14^ It is important to note, however, that most studies have reported mortality risk based on quantiles of carbohydrate intake that are specific to the populations studied. Thus, the effects of carbohydrate intake can depend on the internal reference range for a given population. Furthermore, most analyses of carbohydrate intake have not accounted for the potential effects of specific food sources (ie, animal-based versus plant-based) that are used to replace carbohydrate intake in low carbohydrate intake settings.

Given the need for more evidence to help guide recommendations regarding optimal carbohydrate intake, we did a population-based study of overall carbohydrate consumption, allowing for the possibility of non-linear relationships. Specifically, we investigated the association of carbohydrate intake with mortality and residual lifespan in a large bi-racial cohort of adults living in four US communities, and then combined these mortality data with previous data as part of a meta-analysis to contextualise our findings. We then studied whether the replacement of carbohydrate for animal-based or plant-based sources of fat and protein modified any observed associations.

## Methods

### Study design and participants

The Atherosclerosis Risk in Communities (ARIC) study is an ongoing, prospective observational study of cardiovascular risk factors in four US communities (Forsyth County, NC; Jackson, MS; suburbs of Minneapolis, MN; and Washington County, MD), initially consisting of participants aged 45–64 years who were recruited between 1987 and 1989 (Visit 1).^15^ Study participants were examined at follow-up visits, with the second visit occurring between 1990 and 1992, the third between 1993 and 1995, the fourth between 1996 and 1998, the fifth between 2011 and 2013, and the sixth between 2016 and 2017. At each participating site, an institutional review board approved the study protocol. Participants provided written informed consent at each examination. We excluded participants without complete dietary information or with extreme caloric intake (defined as <600 kcal or >4200 kcal per day for men and <500 kcal or >3600 kcal per day for women).

### Procedures

Participants completed an interview that included a 66-item semi-quantitative food frequency questionnaire (FFQ), modified from a 61-item FFQ designed and validated by Willett and colleagues,^16^ at Visit 1 (1987–89) and Visit 3 (1993–95). Participants reported the frequency with which they consumed particular foods and beverages in nine standard frequency categories (extending from never or less than one time per month, to six or more times per day). Standard portion sizes were provided as a reference for intake estimation, and pictures and food models were shown to the participants by the interviewer at each examination. We used the Harvard Nutrient Database to derive nutrient intakes from the FFQ responses.^16^

### Outcomes

The primary outcome was all-cause mortality, subsequent to the first visit, until the end of 2013. Number of deaths was determined with annual (or later, semi-annual) telephone calls, linkage to local hospital and state health department records, or for those lost to follow-up, linkage to the National Death Index.

### Statistical analysis

We analysed the covariates of age, sex, race (self-reported), study centre, education level (grade school, high school without diploma, high school graduate, vocational school, college graduate, graduate school or professional school), cigarette smoking status (current, former, never), physical activity level (sport and exercise activity and non-sport activity during leisure from Baecke questionnaire^17^), total energy intake (kcal), ARIC test centre location, and diabetes status (defined on the basis of use of anti-diabetic medications, self-report of a physician diagnosis, fasting glucose value ≥126 mg/dL or a non-fasting glucose of ≥200).

We tested the association of baseline characteristics of the ARIC cohort with quantiles of total energy from carbohydrate using linear regression and χ^2^ tests for categorical variables (adjusting for age and sex). We used Cox proportional hazards regression models to calculate hazard ratios (HRs), to quantify the association between carbohydrate intake and the risk of death. We used restricted cubic splines^18^ with 4 knots to express the potentially non-linear association between total energy from carbohydrate intake at Visit 1 and all-cause mortality. We adjusted the ARIC analyses for demographics (age, sex, self-reported race), energy intake (kcal per day), study centre, education, exercise during leisure activity, income level, cigarette smoking, and diabetes. We did a time-varying sensitivity analysis: between baseline ARIC Visit 1 and Visit 3, carbohydrate intake was calculated on the basis of responses from the baseline FFQ. From Visit 3 onwards, the cumulative average of carbohydrate intake was calculated on the basis of the mean of baseline and Visit 3 FFQ responses. We did not update carbohydrate exposures of participants that developed heart disease, diabetes, and stroke before Visit 3, to reduce potential confounding from changes in diet that could arise from the diagnosis of these diseases. We did a mean residual lifetime analysis using previously published methods.^19^ We created actuarial estimates of the age-specific probabilities of death according to each category of carbohydrate intake exposure, and used these estimates to obtain non-parametric age-based Kaplan-Meier estimates of the survival curve for participants at each year of age in each carbohydrate intake category (>65%, 55–65%, 50–55%, 40–50%, 30–40%, and <30%). The expected residual years of survival were estimated as the area under the survival curve up to a maximum age of 93 years. We chose a reference group of 50–55% for the analysis and we did a post-hoc sensitivity analysis using a reference group of 50–60%. We updated the previously published meta-analysis (including papers published between Sept 12, 2012, when the previous meta-analysis ended, and Sept 1, 2017) using previously described methods.^12^ Briefly, papers were eligible for inclusion if they were a published full-text report, observational study, or randomised controlled trial with a minimum of 1 year follow-up, reporting relative risks (ie, HRs, risk ratios, or odds ratios with CIs), and adjusted for at least three of the following factors: age, sex, obesity, smoking status, diabetes, hypertension, hypercholesterolaemia, history of cardio-vascular disease, and family history of cardiovascular disease. We assessed the quality of reports in reference to the CONSORT statement^20^ and the STROBE statement.^21^ We further assessed quality using the Newcastle-Ottawa Scale,^22^ with a score of 5 or less (out of 8) suggesting a high risk of bias. If more than a single study published data from the same cohort with the same endpoint of all-cause mortality, we included the report representing the most inclusive information on the population to avoid overlap. We calculated pooled HRs with 95% CIs using a random-effects model with inverse-variance weighting. We recreated the mortality versus percentage of energy from carbohydrate spline from the PURE study^13^ by extracting published coordinates; we overlaid ARIC data on the graph using an identical reference point of 46·4% kcal from carbohydrate, and closely matched the covariates available in the ARIC cohort with those used in the PURE study, including waist-to-hip ratio.

We created animal-based and plant-based scores by dividing participants into deciles for either animal-derived or plant-derived fat and protein, and carbohydrate intake, expressed as a percentage of energy as previously described.^23,24^ For carbohydrate, participants in the lowest decile received 10 points, whereas participants in the highest decile received 1 point. The order was reversed for animal-derived or plant-derived fat and protein, so that the highest score represented low carbohydrate and high animal-derived or plant-derived fat and protein intake. We used restricted cubic splines to determine the association of all-cause mortality with animal-based and plant-based scores. For meta-analysis of animal and plant-based scores, we calculated pooled HRs with 95% CIs using a random-effects model with inverse-variance weighting for those cohorts that had these data available.

In post-hoc sensitivity analyses, we explored the outcome of cardiovascular death (defined using International Classification of Diseases [ICD]-9 codes 390–459 and ICD-10 codes I00–I99).

## Results

Baseline characteristics of the ARIC study population, according to quantiles of percentage energy from carbohydrate intake, are shown in table 1. Mean carbohydrate intake was 48·9% (SD 9·4). Participants who consumed a relatively low percentage of total energy from carbohydrates (ie, participants in the lowest quantiles) were more likely to be young, male, a self-reported race other than black, college graduates, have high body-mass index, exercise less during leisure time, have high household income, smoke cigarettes, and have diabetes. Overall, mean consumption of energy from animal fat and protein was higher than from plant fat and protein across all carbohydrate quantiles (table 1). Participants in the lowest carbohydrate quantile had higher average consumption of animal fat and protein and lower average consumption of plant protein and dietary fibre than participants in the other quantiles. Plant fat and total energy intake had reverse U-shaped or J-shaped relationships across carbohydrate quantiles: participants in both the first and fifth quantile had lower mean plant-derived fats and calorie consumption compared with those in the intermediate quantiles (table 1). Prevalence of hypertension was similar across carbohydrate quantiles. There was no significant difference in weight gain at 3-year or 6-year timepoints across carbohydrate quantiles (table 1).

**Figure 1:**
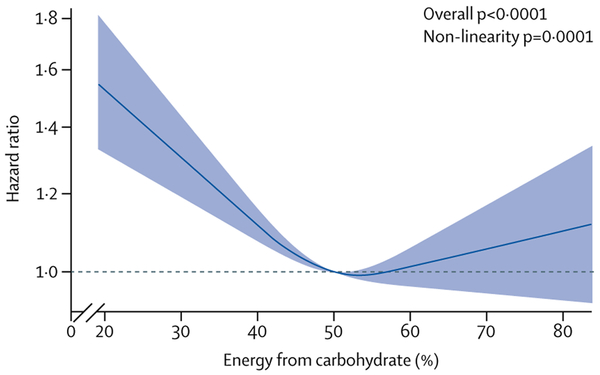
U-shaped association between percentage of energy from carbohydrate and all-cause mortality in the ARIC cohort The reference level is 50% energy from carbohydrate. Results are adjusted for age, sex, race, ARIC test centre, total energy consumption, diabetes, cigarette smoking, physical activity, income level, and education. ARIC=Atherosclerosis Risk in Communities.

The median length of follow-up was 25 years, during which there were 6283 deaths. The highest risk of mortality was observed in participants with the lowest carbohydrate consumption, in both unadjusted and adjusted models (p<0·001; figure 1; appendix p 8). However, the relationship between carbohydrate consumption and risk of mortality was significantly nonlinear (p<0·001), resulting in a U-shaped association, with the lowest observed risk associated with carbohydrate consumption of 50–55% (figure 1). There were corresponding significant differences in mean residual lifespan based on carbohydrate intake (appendix p 2). For example, we estimated that a 50-year-old participant with intake of less than 30% of energy from carbohydrate would have a projected life expectancy of 29·1 years, compared with 33·1 years for a participant who consumed 50–55% of energy from carbohydrate (difference 4·0 years [95% CI 2·6, 5·3]). Similarly, we estimated that a 50-year-old participant with high carbohydrate intake (>65% of energy from carbohydrate) would have a projected life expectancy of 32·0 years, compared with 33·1 years for a participant who consumed 50–55% of energy from carbohydrate (difference 1·1 years [0·1, 2·0]). We did a sensitivity analysis using 50–60% energy from carbohydrate as the comparison group, with similar findings (data not shown). The association of overall carbohydrate intake with cardiovascular and non-cardiovascular mortality is shown in the appendix (pp 3, 4). There were similar results when we used dietary information from Visit 1 and Visit 3 in the sensitivity analysis (appendix pp 5, 6).

**Figure 2:**
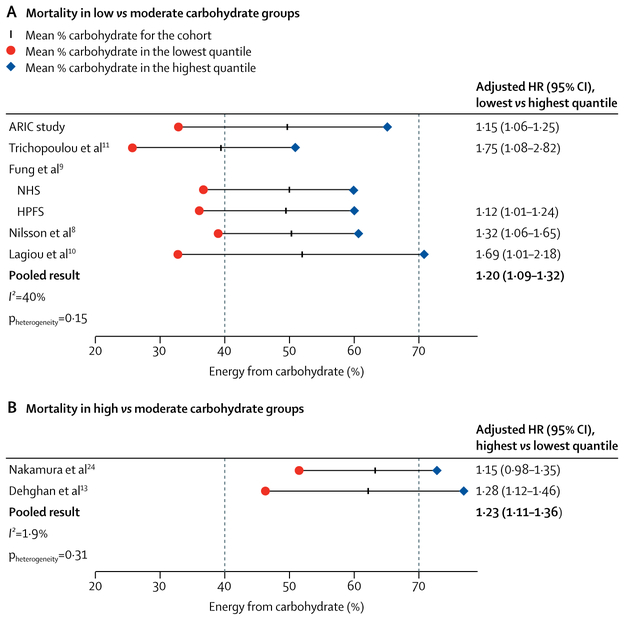
Carbohydrate intake and mortality risk across multiple cohort studies Mean values of percentage of energy from carbohydrate (% carbohydrate) reported in previously studied cohorts from lowest to highest quantiles. Adjusted HRs are from analyses of low carbohydrate scores versus high carbohydrate scores (n=432179, all-cause deaths=40 181). Dotted lines indicate cutoffs for low carbohydrate (<40%) and high carbohydrate (>70%). (A) Low carbohydrate versus moderate carbohydrate (40–70%) reference group. (B) High carbohydrate versus moderate carbohydrate reference group. HR=hazard ratio. ARIC=Atherosclerosis Risk in Communities. NHS=Nurses Health Study. HPFS=Health Professionals Follow-up Study.

We updated a meta-analysis^12^ published in 2012, by identifying two additional studies that had since been published and that met inclusion criteria, using previously defined methods;^13,24^ we also added results from ARIC because they met previously defined inclusion criteria^12^ (table 2). Including data from the ARIC cohort, there were 432 179 participants in eight cohort studies investigating carbohydrate intake, with 40 181 (9·3%) deaths reported. Because there was significantly lower consumption of carbohydrate in European and North American regions compared with Asian countries, low-income countries, and multinational cohorts (p<0·001), studies fell into two categories in the meta-analysis: North American and European studies (mean carbohydrate intake approximately 50%) that compared low carbohydrate diets with primarily moderate carbohydrate consumption as the reference (figure 2A), and Asian and multinational studies (mean carbohydrate intake approximately 61%) that compared high carbohydrate consumption with moderate carbohydrate consumption as the reference (figure 2B; table 2). The association between carbohydrate consumption and mortality was dependent on the range of carbohydrate intake. Figure 2 illustrates the significantly increased risk of all-cause mortality among participants with low carbohydrate versus moderate carbohydrate consumption (pooled HR 1·20, 95% CI 1·09–1·32; p<0·0001). This relationship remained significant if the ARIC study was excluded from the analysis (1·31, 1·07–1·58; p=0·007). High carbohydrate consumption was associated with a significantly higher risk of all-cause mortality compared with moderate carbohydrate consumption (1**·**23, 1**·**11–1**·**36; p<0·0001; figure 2).

**Figure 3:**
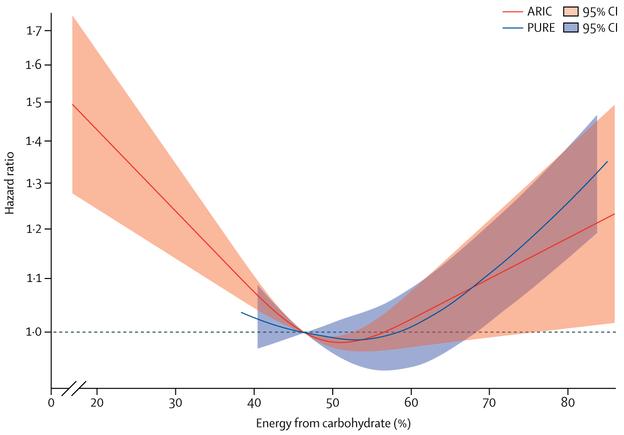
U-shaped association between percentage of energy from carbohydrate and all-cause mortality in the ARIC and PURE cohort studies The reference level is 46·4% energy from carbohydrate. ARIC results are adjusted for age, sex, education, waist-to-hip ratio, smoking, physical activity, diabetes, ARIC test centre, and energy intake. PURE results are are adjusted for age, sex, education, waist-to-hip ratio, smoking, physical activity, diabetes, urban or rural location, centre, geographical regions, and energy intake.^13^ The mean percentage of energy from carbohydrate in ARIC is 49%, and from PURE it is 61%. ARIC=Atherosclerosis Risk in Communities. PURE=Prospective Urban Rural Epidemiology.

The ARIC and PURE studies were the only two cohorts for which data were published or available about the continuous percentage of energy from carbohydrate. Figure 3 shows the overlapping and continuous relationship between percentage of energy from carbohydrate intake and mortality in these cohorts. By comparison with ARIC, the PURE study^13^ assessed participants primarily at the high end of the overall range of percentage of energy from carbohydrate consumption (figures 2, 3). However, the associations between primarily high carbohydrate intake and mortality in the PURE study still fell within the confidence intervals of those observed in ARIC (figure 3).

To explore the association between mortality and the source of fat and protein alternatives to carbohydrate intake, we compared studies that assessed animal-based and plant-based scores, which represented increasing substitution of animal-based or plant-based fat and protein for carbohydrate intake (table 2). Baseline characteristics of the ARIC study population, according to animal-based or plant-based low carbohydrate diet scores, are shown in the appendix (pp 9–11). The plant-based low carbohydrate dietary score was associated with higher average intake of vegetables but lower fruit intake (appendix p 11). By contrast, the animal-based low carbohydrate dietary score was associated with lower average intake of both fruit and vegetables (appendix pp 9, 10). Both low carbohydrate diets were associated with higher fat intake in exchange for carbohydrate, although the plant-based low carbohydrate diet had higher average polyunsaturated fat and lower saturated fat intake compared with the animal-based low carbohydrate diet (appendix pp 9–11). Overall, total protein intake was higher in the animal-based diet (appendix p 9). We determined the five foods that differed most significantly between the highest and lowest quantiles of animal-based and plant-based low carbohydrate dietary score. The animal-based low carbohydrate diet had more servings per day than did higher carbohydrate diets of beef, pork, and lamb as the main dish; beef, pork, and lamb as a side dish; chicken with the skin on; chicken with the skin off; and cheese (appendix p 10). The plant-based low carbohydrate diet had more servings per day of nuts, peanut butter, dark or grain breads, chocolate, and white bread than did higher carbohydrate diets (appendix p 11). Both low carbohydrate diets were lower in average regular soft drink intake (appendix pp 10, 11). In the ARIC cohort and in meta-analysis, increased consumption of animal-based protein and fat instead of carbohydrate was associated with a significant increase in all-cause mortality (p<0·0001; table 3). Alternatively, increased consumption of plant-based protein and fat instead of carbohydrate was associated with a significant decrease in all-cause mortality (p<0·0001; table 3). The animal and plant-based findings were consistent for cardiovascular and non-cardiovascular mortality (appendix pp 3, 4). Sensitivity analysis of plant-specific and animal-specific findings, using dietary information from Visit 1 and Visit 3, yielded similar results (appendix pp 6, 7). Similarly, in the meta-analysis, mortality increased when animal-derived fat and protein were substituted for carbohydrate, and decreased when these substitutions were plant-based (table 3). In the post-hoc sensitivity analysis, we assessed all meta-analyses using a fixedeffects model, with similar findings. Additionally, to minimise the likelihood of reverse causation, we did a sensitivity analysis whereby individuals with cardiovascular disease, diabetes, or cancer at baseline were excluded from the analyses. These post-hoc analyses also yielded similar results.

## Discussion

In a large cohort of adults living in four diverse US communities, with more than two decades of follow-up, mid-life dietary patterns marked by both low carbohydrate (<40% of energy from carbohydrate) and high carbohydrate (>70% of energy from carbohydrate) consumption were associated with increased mortality risk and shorter residual lifespan, with minimum risk observed with 50–55% of energy from carbohydrate. These findings reflect a U-shaped relationship between carbohydrate intake and mortality, and were corroborated by data from other North American, European, Asian and multinational cohorts, combined as part of a meta-analysis. However, low carbohydrate dietary patterns that replaced energy from carbohydrate with energy from animal-derived protein or fat were associated with greater risk. However, this association was reversed when energy from carbohydrate was replaced with plant-derived protein or fat.

In this study, the association of carbohydrate intake with mortality was dependent on the range of carbohydrate intake. The range of carbohydrate intake differs by geographical and socioeconomic factors; percentage of energy from carbohydrates have been lower in North American and European cohort studies (mean values generally ≤50%) than in Asian or multinational cohorts, which are largely comprised of low-income and middle-income countries (mean values >60%). Overall, there was a U-shaped relationship between carbohydrate intake and mortality, but the North American and European cohorts primarily represented the left side of the U-shaped curve whereas Asian and less economically advanced nations (as included in the PURE study) represented the right side of the curve. North American and European cohort studies have compared true low carbohydrate dietary patterns (in terms of absolute value of <40% of total energy intake) and consistently found a modest yet significantly increased relative risk of all-cause death when compared with the highest quantile (generally still falling in a moderate carbohydrate range of 40–70%). The NIPPON DATA80^24^ and PURE^13^ studies represent the right side of the curve for absolute intake of percentage of energy from carbohydrate, and consistently show a modest yet significantly decreased relative risk of all-cause death when comparing moderate (45–55% of total energy) to the highest quantile (>70% of total energy).

Findings from this study suggest that previous analyses of carbohydrate intake that focused on quantiles of consumption and then searched for a trend across those quantiles seem to have overlooked valuable information. Using the carbohydrate intake data continuously provides more granular information and allowed us to identify a more U-shaped relationship between carbohydrate consumption and risk, which might otherwise not have been evident. Continuous data have not been published for North American or European cohorts; several previous studies only showed a linear relationship,^8,10,11^ whereas others that reported quantiles were suggestive of U-shaped or J-shaped relationships.^9,24^ The relationship between dietary carbohydrate and mortality was reported as a continuous relationship in the PURE study, with intake ranging primarily from moderate to high carbohydrate, but still fell within the confidence intervals of what we observed in ARIC, with intake ranging primarily from low to moderate carbohydrate, further supporting a U-shaped relationship between carbohydrate intake and mortality. Although this study included quantile-based analyses to the extent that previous work has used such analyses, and we illustrate how the ARIC data fit in that context, the continuous analyses probably reflect a much closer representation of the true relationship between carbohydrate intake and mortality.

To further examine the potential effects of protein and fat sources supplanting carbohydrate intake, we investigated animal-based and plant-based diets in the ARIC cohort. We found that low carbohydrate dietary patterns favouring animal-derived protein and fat sources were associated with higher mortality, in accordance with results from the Nurses’ Health Study and Health Professionals Follow up Study.^9^ However, low carbohydrate diets that favoured plant-derived protein and fat intake were associated with lower mortality, also consistent with previous results.^9,24^ These data suggest that the source of the protein and fat substituted for carbohydrates in the diet might notably modify the relationship between carbohydrate intake and mortality. Previous work has shown a less consistent relationship between overall carbohydrate intake and cardiovascular death by comparison with all-cause mortality.^12^ However, in our analysis, when carbohydrate is substituted for higher animal fat or protein intake it is associated with both higher cardiovascular and non-cardiovascular death, whereas plant-based substitutions are associated with both lower cardiovascular and non-cardiovascular death, indicating that food source could be an important consideration for both causes of mortality.

There are several possible explanations for our main findings. Low carbohydrate diets have tended to result in lower intake of vegetables, fruits, and grains and increased intakes of protein from animal sources,^23,25–27^ as observed in the ARIC cohort, which has been associated with higher mortality. It is likely that different amounts of bioactive dietary components in low carbohydrate versus balanced diets, such as branched-chain amino acids, fatty acids, fibre, phytochemicals, haem iron, and vitamins and minerals are involved.^28^ Long-term effects of a low carbohydrate diet with typically low plant and increased animal protein and fat consumption have been hypothesised to stimulate inflammatory pathways, biological ageing, and oxidative stress. On the other end of the spectrum, high carbohydrate diets, which are common in Asian and less economically advantaged nations, tend to be high in refined carbohydrates, such as white rice; these types of diets might reflect poor food quality^13,24^ and confer a chronically high glycaemic load that can lead to negative metabolic consequences.^29^

There are limitations to this study that merit consideration. This study represents observational data and is not a clinical trial; however, randomised trials of low carbohydrate diets on mortality are not practical because of the long duration of study required. Another limitation of this study is that diet was only assessed at two time intervals, spanning a 6-year period, and dietary patterns could change during 25 years. However, because participants are able to increase or decrease their consumption of carbohydrates during the course of follow-up, any dietary changes that occur after the described assessments would be expected to attenuate any observed associations. Our conclusions about animal fat and protein might have less generalisability to Asian cultures, which often feature very high carbohydrate consumption but with a primary meat source that is often from fish. In fact, the plant score calculated in the Japanese cohort, NIPPON DATA80,^24^ included fish as a source of protein as well. Hence, animal scores reported here are composed largely of beef, pork, and fowl, in addition to fish. An additional limitation is that the international data^13^ about very high carbohydrate intakes, largely derived from China, are, on average, higher than the national data,^30^ for unclear reasons. However, the advantage of these data is that they include multi-racial and ethnic groups across a spectrum of socioeconomic groups, and they are representative of many high quality cohorts. Given the relatively small number of individuals adhering to low carbohydrate diets with mainly plant-based protein and fat sources of macronutrients, this study could not definitively examine the relative benefits of this diet compared with other dietary patterns. Our study focused on general carbohydrate intake, which represents a heterogeneous group of dietary components. Any number and combination of dietary components could have been considered and adjusted for in this analysis; therefore, some confounders might have been unadjusted for. Ideally, it would be preferable to do an individual-level meta-analysis in a collaborative effort that would have allowed for consistent adjustment for confounders in pooled analysis. Finally, some degree of measurement error is unavoidable for all dietary assessment methods, and the absolute intakes need to be interpreted cautiously.

Our findings suggest a negative long-term association between life expectancy and both low carbohydrate and high carbohydrate diets when food sources are not taken into account. These data also provide further evidence that animal-based low carbohydrate diets should be discouraged. Alternatively, when restricting carbohydrate intake, replacement of carbohydrates with predominantly plant-based fats and proteins could be considered as a long-term approach to promote healthy ageing.

## Supplementary Material

1

## Figures and Tables

**Table 1:** Population characteristics in the Atherosclerosis Risk in Communities study, by quantile

	Q1 (n=3086)	Q2 (n=3086)	Q3 (n=3085)	Q4 (n=3086)	Q5 (n=3085)	p_trend_
Median % of energy from carbohydrate	37% (5.7)	44% (2.5)	49% (2.2)	53% (2.8)	61% (6.3)	NA
Mean age, years (SD)	53.7 (5.7)	54.3 (5.7)	54.3 (5.8)	54.3 (5.8)	54.3 (5.8)	<0.0001
Sex						<0.0001
Men	1635 (53%)	1496 (48%)	1379 (45%)	1294 (42%)	1112 (36%)	··
Women	1451 (47%)	1590 (52%)	1706 (55%)	1792 (58%)	1973 (64%)	··
Race						<0.0001
White	2345 (76%)	2320 (75%)	2255 (73%)	2203 (71%)	2133 (69%)	··
Black	731 (24%)	764 (25%)	822 (27%)	875 (28%)	932 (30%)	··
Asian	4 (<1%)	1 (<1%)	6 (<1%)	6 (<1%)	17 (1%)	··
Native American	6 (<1%)	1 (<1%)	2 (<1%)	2 (<1%)	3 (<1%)	··
Mean BMI, kg/m^2^	28.0 (0.1)	27.9 (0.1)	27.6 (0.1)	27.6 (0.1)	27.4 (0.1)	<0.0001
Diabetes	415 (13%)	404 (13%)	345 (11%)	330 (11%)	316 (10%)	<0.0001
Hypertension	1095 (35%)	1028 (33%)	1046 (34%)	1052 (34%)	1148 (37%)	0.4436
Smoking^*^						<0.0001
Current smoker	1016/3083 (33%)	821/3085 (27%)	787/3083 (26%)	707/3084 (23%)	687/3084 (22%)	··
Former smoker	1079/3083 (35%)	1042/3085 (34%)	995/3083 (32%)	950/3084 (31%)	899/3084 (29%)	··
Never smoker	988/3083 (32%)	1220/3085 (40%)	1301/3083 (42%)	1427/3084 (46%)	1496/3084 (48%)	··
Unknown	0	2/3085 (<1%)	0	0	2/3084 (<1%)	··
Highest exercise activity (quantile 5)	474 (15%)	534 (17%)	575 (19%)	581 (19%)	614 (20%)	<0.0001
College graduates	905 (29%)	860 (28%)	774 (25%)	738 (24%)	674 (22%)	<0.0001
Household income^*^						<0.0001
<$5000	154/2909 (5%)	138/2913 (5%)	154/2918 (5%)	154/2905 (5%)	174/2876 (6%)	··
$5000–$7999	118/2909 (4%)	107/2913 (4%)	108/2918 (4%)	125/2905 (4%)	164/2876 (6%)	··
$8000–$11 999	140/2909 (5%)	160/2913 (5%)	187/2918 (6%)	187/2905 (6%)	192/2876 (7%)	··
$12 000–$15 999	185/2909 (6%)	203/2913 (7%)	205/2918 (7%)	229/2905 (8%)	239/2876 (8%)	··
$16 000–$24 999	406/2909 (14%)	385/2913 (13%)	453/2918 (16%)	462/2905 (16%)	480/2876 (17%)	··
$25 000–$34 999	456/2909 (16%)	531/2913 (18%)	524/2918 (18%)	529/2905 (18%)	553/2876 (19%)	··
$35 000–$49 999	582/2909 (20%)	587/2913 (20%)	584/2918 (20%)	558/2905 (19%)	507/2876 (18%)	··
>$50 000	868/2909 (30%)	802/2913 (28%)	703/2918 (24%)	661/2905 (23%)	567/2876 (20%)	··
Mean total energy intake, kcal	1558 (11)	1655 (11)	1660 (11)	1646 (11)	1607 (11)	0.0092
Mean animal protein % of energy	16.9% (0.1)	14.8% (0·1)	13.5% (0.1)	12.3% (0.1)	10.1% (0.1)	<0.0001
Mean plant protein % of energy	3.9% (0.02)	4.3% (0·02)	4.5% (0.02)	4.6% (0.02)	4.8% (0.02)	<0.0001
Mean animal fat % of energy	26.3% (0.1)	22.4% (0·1)	19.9% (0.1)	17.6% (0.1)	13.6% (0.1)	<0.0001
Mean plant fat % of energy	12.5% (0.1)	13.6% (0·1)	13.6% (0.1)	13.2% (0.1)	11.5% (0.1)	<0.0001
Mean dietary fibre, g	13.5 (0.1)	16.5 (0.1)	17.7 (0.1)	18.7 (0.1)	19.8 (0.1)	<0.0001
Glycaemic index	71·8 (0·1)	74.1 (0.1)	74.9 (0.1)	76.0 (0.1)	76.7 (0.1)	<0.0001
Glycaemic load	100.6 (1.1)	134.6 (1.1)	151.1 (1.1)	166.8 (1.1)	191.7 (1.1)	<0.0001
Change in BMI						
3-year change	0.36 (0.03)	0.33 (0.03)	0.31 (0.03)	0.32 (0.03)	0.41 (0.03)	0.3878
6-year change	0.94 (0.04)	0.93 (0.04)	0.86 (0.04)	0.94 (0.04)	0.92 (0.04)	0.8206

Data are median (IQR), mean (SE), n (%), or n/N (%), unless otherwise indicated. Standard errors are provided for age-adjusted and sex-adjusted values. Baseline characteristics are from the study population (n=15428) at baseline Visit 1 (1987–89), according to quantiles of percentage of energy from carbohydrate adjusted for age and sex. Income is reported in US$. NA=not applicable. BMI=body-mass index.

* Some missing values for this category.

**Table 2:** Meta-analysis study characteristics

	Cohort	Country or region	Follow-up, years	Total number of participant (proportion of women)	Age, years	Proportion of patients with diabetes	Proportion of patients with previous cardiovascular disease	Animal and plant score	All-cause death (n)
This study	ARIC	USA	25 (median)	15 428 (56%)	45–64	12%	4%	Yes	6283
Lagiou et al^10^	Scandinavian Women’s Lifestyle and Health Cohort	Sweden	12 (mean)	42 237 (100%)	30–49	Patients excluded	Patients excluded	No	588
Trichopoulou et al^11^	EPIC	Greece	4.9 (mean)	22 944 (59%)	20–86	Patients excluded	Patients excluded	No	455
Fung et al^9^	NHS	USA	26	85 168 (100%)	34–59	Patients excluded	Patients excluded	Yes	12 555
Fung et al^9^	HPFS	USA	20	44 548 (0%)	40–75	Patients excluded	Patients excluded	Yes	8678
Nilsson et al^8^	Västerbotten Intervention Programme	Sweden	10 (median)	77 319 (51%)	49 (median)	3%	NR	No	2383
Nakamura et al^24^	NIPPON DATA80	Japan	29	9200 (56%)	51 (mean)	NR	NR	Yes	3443
Dehghan et al^13^	PURE	Multinational	7.4 (median)	135 335 (58%)	50.3 (mean)	7.1%	NR	No	5796

ARIC=Atherosclerosis Risk in Communities. EPIC=European Prospective Investigation into Cancer and Nutrition. NHS=Nurses Health Study. HPFS=Health Professionals Follow-up Study. NR=not recorded. NIPPON DATA80 National Integrated Project for Prospective Observation of Non-communicable Disease and its Trends in the Aged. PURE=Prospective Urban Rural Epidemiology.

**Table 3:** Association between diets that substitute carbohydrates for animal-based or plant-based protein and fat with mortality in multiple cohort studies

Study		HR (95% CI)
**Substitution of carbohydrate for animal protein and fat**		
Low-to-moderate carbohydrate consumption	Fung et al^9^ (HPFS)	1.31 (1.19–1.44)
Low-to-moderate carbohydrate consumption	Fung et al^9^ (NHS)	1.17 (1.08–1.26)
Low-to-moderate carbohydrate consumption	ARIC	1.20 (1.09–1.32)
Low-to-moderate carbohydrate consumption	Combined low-to-moderate cohorts	1.22 (1.14–1.31)
Moderate-to-high carbohydrate consumption	Nakamura et al^24^	1.00 (0.87–1.19)
Meta-analysis (pooled result)	..	1.18 (1.08–1.29); p<0.0001
**Substitution of carbohydrate for plant protein and fat**		
Low-to-moderate carbohydrate consumption	Fung et al^9^ (HPFS)	0.81 (0.74–0.89)
Low-to-moderate carbohydrate consumption	Fung et al^9^ (NHS)	0.79 (0.73–0.85)
Low-to-moderate carbohydrate consumption	ARIC	0.86 (0.75–0.99)
Low-to-moderate carbohydrate consumption	Combined low-to-moderate cohorts	0.81 (0.76–0.85)
Moderate-to-high carbohydrate consumption	Nakamura et al^24^	0.92 (0.80–1.09)
Meta-analysis (pooled result)	..	0.82 (0.78–0.87); p<0.0001

Data are for 154 344 participants and 30 959 deaths. HR=hazard ratio. HPFS=Health Professionals Follow-up Study. NHS=Nurses Health Study. ARIC=Atherosclerosis Risk in Communities.

## Evidence before this study

Although many randomised controlled trials of low carbohydrate diets suggest beneficial short-term weight loss and improvements in cardiometabolic risk, mortality risk has typically not been investigated in light of the practical challenges posed by studies involving very long durations of follow-up. Data from large prospective cohorts have been used to estimate the long-term health effects of low carbohydrate diets, but have generated conflicting results. For instance, the meta-analysis of large cohort studies in North America and Europe has suggested increased mortality associated with low carbohydrate intake. Conversely, recently published multinational and Asian studies have reported increased mortality in association with high carbohydrate intake. Most previous studies have reported the risk of mortality on the basis of the range of carbohydrate intake specific to the population studied, thus limiting both interpretability and generalisability. Furthermore, many previous studies of carbohydrate intake have not accounted for the potential effects of food source. Weupdated a previously published meta-analysis by searching MEDLINE, Embase, ISI Web of Science, Cochrane Library, and ClinicalTrials.gov, using a combination of the keywords “lowcarbohydrate diet” OR “carbohydrate-restricted diet”, AND “mortality” OR “survival”, to identify relevant publications published between Sept 12, 2012, and Sept 1, 2017.

## Added value of this study

We studied a large prospective cohort, with a median follow-up of 25 years, to examine the association of carbohydrate intake with mortality in four US communities from diverse socioeconomic backgrounds. We used statistical methods that allowed for the possibility of non-linear associations. We then contextualised our findings in a meta-analysis, combining these data with those from other North American, European, Asian and multinational cohorts. We identified seven studies in addition to the index cohort (432 179 participants, 40 181 deaths). There was a U-shaped relationship between carbohydrate intake and mortality in the Atherosclerosis Risk in Communities cohort, a finding that was consistent in the meta-analysis combining these data with those from the other cohorts. When assessing total carbohydrate without regard to specific food source, diets with high (>70%) or low (<40%) percentage of energy from carbohydrates were associated with increased mortality, with minimal risk observed between 50–55%. Low carbohydrate dietary patterns that replaced carbohydrate with animal-derived protein or fat were associated with greater mortality risk, whereas this association was inverse when energy from carbohydrate was replaced with plant-derived protein or fat. These findings were also corroborated in the meta-analysis.

## Implications of all the available evidence

Our findings suggest a U-shaped relationship between life expectancy and overall carbohydrate intake, in which lifespan is greatest among people with 50–55% carbohydrate intake, a level that might be considered moderate in North America and Europe but low in other regions, such as Asia. These data provide further evidence that animal-based low carbohydrate diets, which are more prevalent in North American and European populations, should be discouraged. Alternatively, if restricting carbohydrate intake is a chosen approach for weight loss or cardiometabolic risk reduction, replacement of carbohydrates with predominantly plant-based fats and proteins could be considered as a long-term approach to promote healthy ageing.
