# Supplementary material for: Dietary carbohydrate intake and mortality: a prospective cohort study and meta-analysis
Source: Lancet Public Health. Author manuscript; Available in PMC 2019 Jan 20. (PMC6339822; doi:10.1016/S2468-2667(18)30135-X)
Supplement: 1 [file NIHMS989986-supplement-1.pdf]

THE LANCET
Public Health

Supplementary appendix

This appendix formed part of the original submission and has been peer reviewed. We post it as supplied by the authors.

Supplement to: Seidelmann SB, Claggett B, Cheng S, et al. Dietary carbohydrate intake and mortality: a prospective cohort study and meta-analysis. *Lancet Public Health* 2018; published online Aug 16. http://dx.doi.org/10.1016/S2468-2667(18)30135-X.

APPENDIX

Supplementary Figure 1. Probability of all-cause Death by Carbohydrate Intake. Shown are age-based Kaplan–Meier curves for participants in the Atherosclerosis Risk in Communities Study by categories of total energy from carbohydrate (% carb) from the age of 50 years with respect to freedom from all-cause mortality.

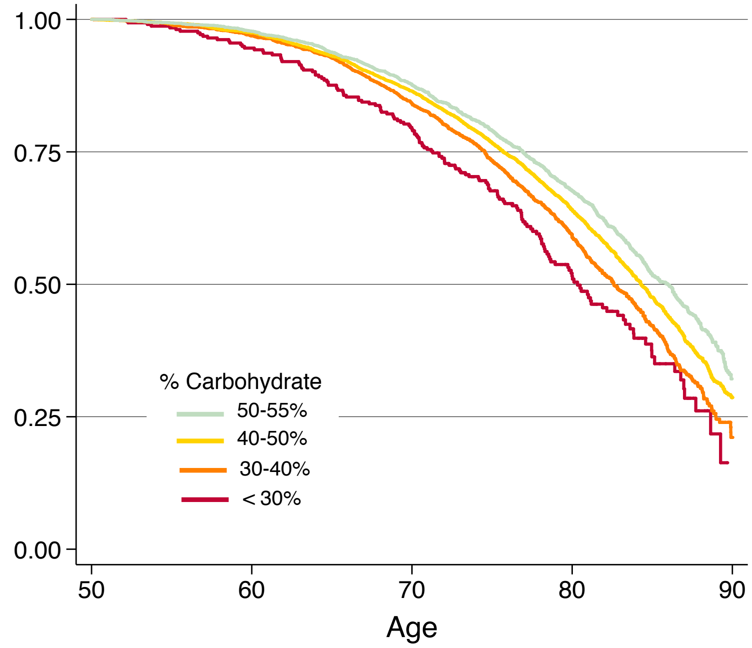

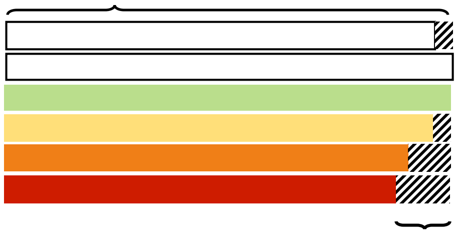

% Carb	Years Difference	P value for difference
>65%	-1.1 (-0.1, -2.0) years	P=0.028
55-65%	0.1 (-0.4, 0.7) years	P=0.7
50 to 55%	Reference=33.1 years	—
40 to 50%	-1.0 (-0.5, -1.5) years	$P=1.2 \times 10^{-4}$
30 to 40%	-2.3 (-1.7, -2.9) years	$P=8.4 \times 10^{-13}$
<30%	-4.0 (-2.6, -5.3) years	$P=1.1 \times 10^{-8}$

Supplementary Figure 2. Association of carbohydrate variants diets with cardiovascular (CV) and non-cardiovascular death. a) Association between percent energy from carbohydrate and CV (red) and non-CV (blue) mortality in the ARIC cohort (N=15,428; 1903 CV and 4380 non-CV deaths). Reference level=50% energy from carbohydrate. Associations between % energy from carbohydrate substituted with Animal (b) or Plant (c) based sources of protein and fat with mortality in the ARIC cohort. Adjusted for age, gender, race, ARIC test center, total energy consumption, diabetes, cigarette smoking, physical activity, income level and education. Reference=population mean; z-scores provided. Dotted lines represent 95% confidence intervals. CV death defined from ICD-9 codes 390-459, or ICD-10 codes I00–I99.

a.

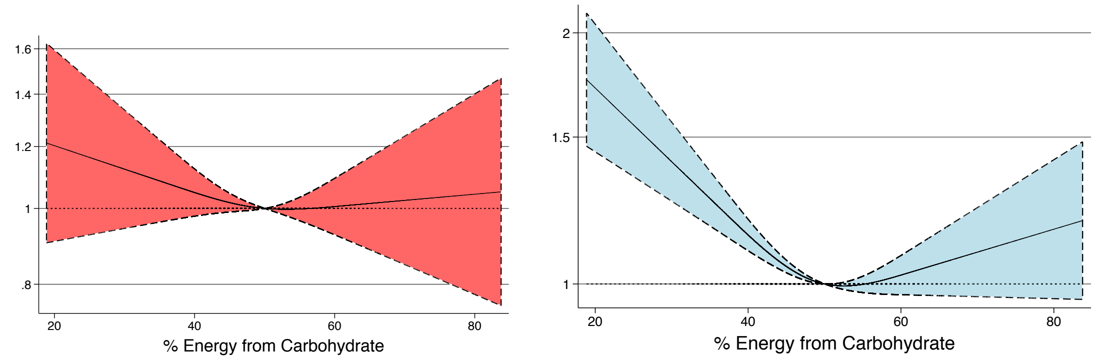
1.6
1.4
1.2
1
.8
20
40
60
80
% Energy from Carbohydrate
2
1.5
1
20
40
60
80
% Energy from Carbohydrate

b.

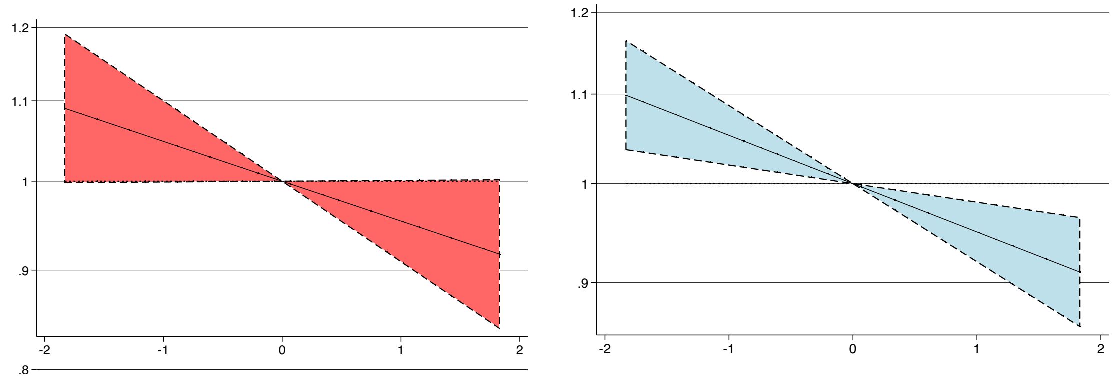
1.2
1.1
1
.9
.8
-2
-1
0
1
2
1.2
1.1
1
.9
-2
-1
0
1
2

c.

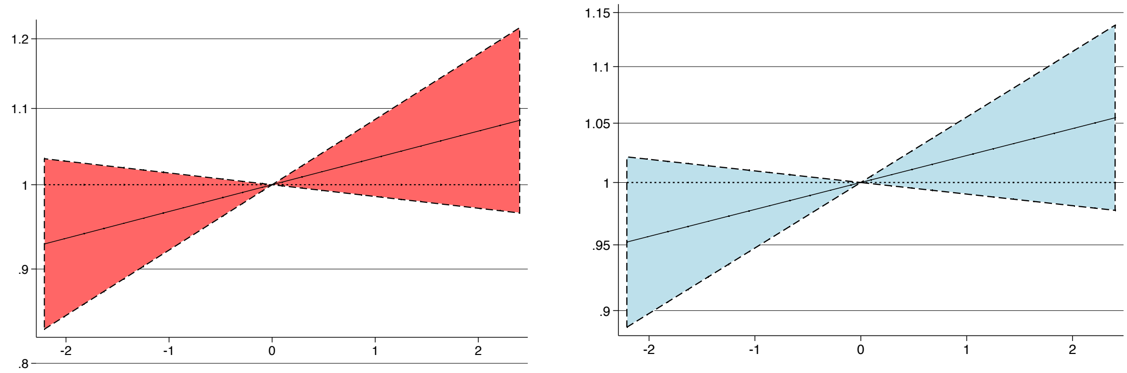
1.2
1.1
1
.9
.8
-2
-1
0
1
2
1.15
1.1
1.05
1
.95
.9
-2
-1
0
1
2

Supplementary Figure 3. Time varying analyses incorporating ARIC visit 1 baseline and visit 3 dietary data. a) U-shaped association between percent energy from carbohydrate and all-cause mortality in the ARIC cohort (N=15,428; 6283 deaths). Reference level=50% energy from carbohydrate. Associations between % energy from carbohydrate substituted with Animal (b) or Plant (c) based sources of protein and fat with mortality. Adjusted for age, gender, race, ARIC test center, total energy consumption, diabetes, cigarette smoking, physical activity, income level and education. Reference=population mean; z-scores provided. Dotted lines represent 95% confidence intervals.

a.

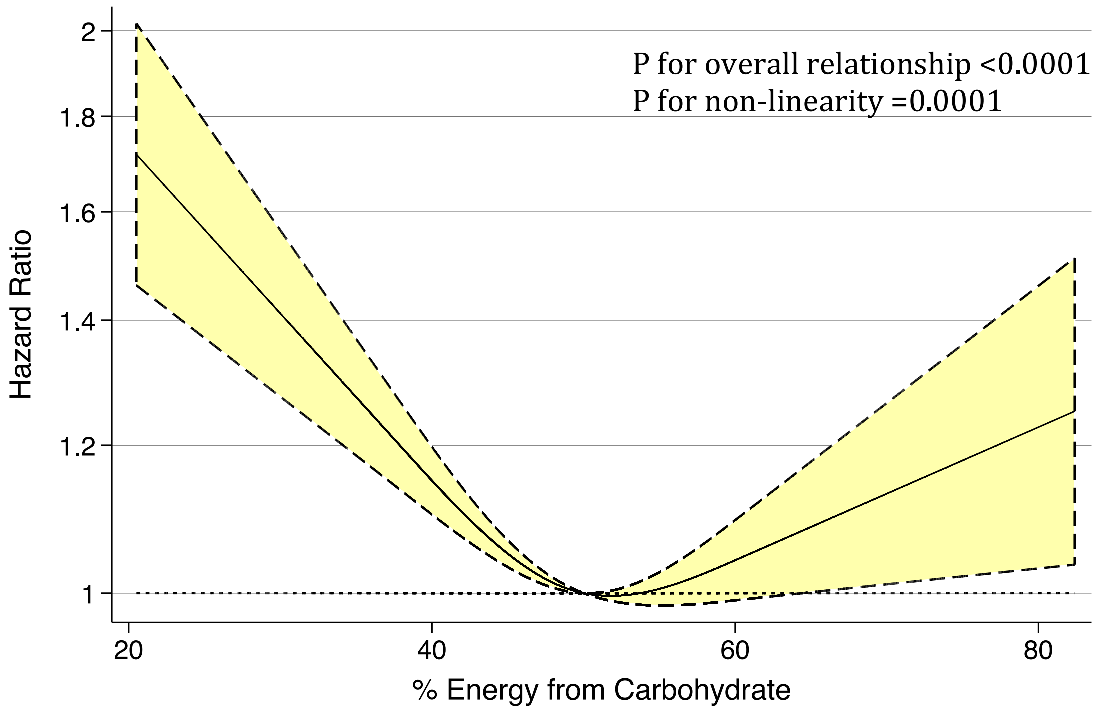

b.

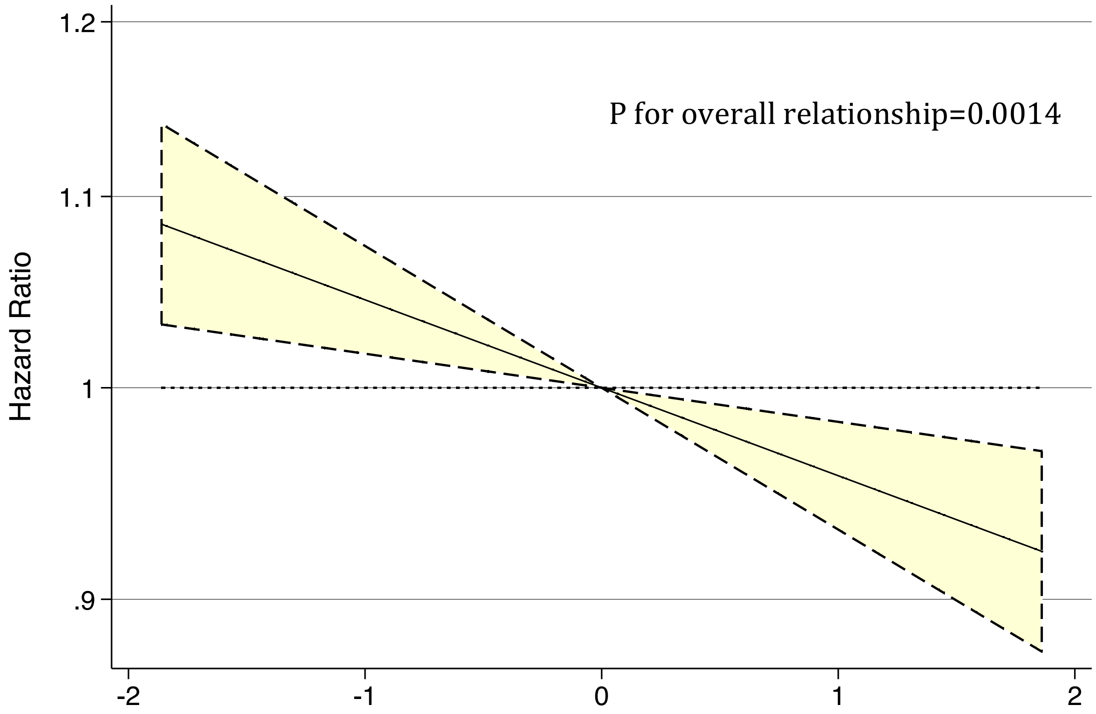

←**High Animal** Fat/Protein **Lower Animal** Fat/Protein→
←**Low Carb**ohydrate **Higher Carb**ohydrate→

c.

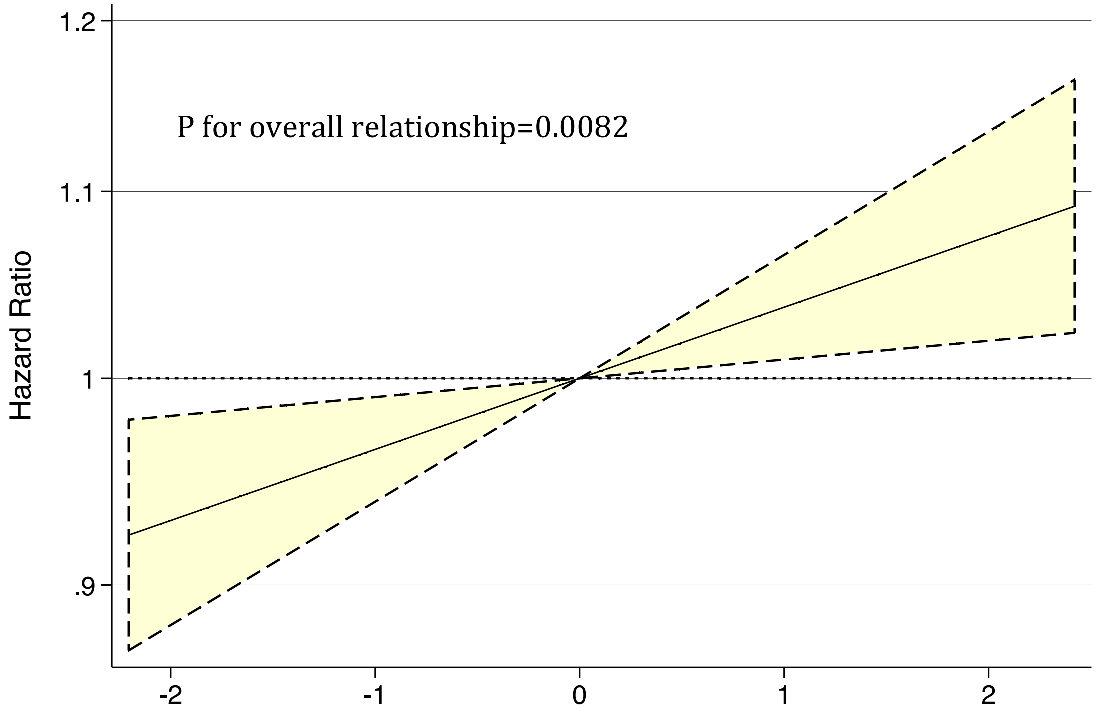

←**Higher Plant** Fat/Protein **Low Plant** Fat/Protein→
←**Low Carb**ohydrate **Higher Carb**ohydrate→

Supplementary Table 1. Percent energy from carbohydrate and all-cause mortality in the ARIC cohort (N=15,428; 6283 deaths). Hazard Ratio (HR) and 95% confidence interval (CI) are reported.

Carbohydrate Range	<30%	30-40%	40-50%	50-55%	55-65%	>65%
N	315	2242	6097	3026	3034	714
Mean %carb, ±SD	26.3 ± 3.7	36.3 ± 2.6	45.4 ± 2.8	52.3 ± 1.4	59.0 ± 2.7	70.0 ± 4.8
Mortality, N(%)	163 (51.7%)	986 (44.0%)	2533 (41.5%)	1162 (38.4%)	1150 (37.9%)	289 (40.5%)
Model 1, HR (95% CI)	1.58 (1.34-1.87)	1.32 (1.21-1.43)	1.12 (1.05-1.21)	Reference	0.99 (0.91-1.07)	1.15 (1.01-1.31)
Model 2, HR (95% CI)	1.37 (1.16-1.63)	1.21 (1.11-1.32)	1.11 (1.03-1.19)	Reference	1.01 (0.93-1.10)	1.16 (1.02-1.33)

Model 1 adjusted for age, race and gender. Model 2 adjusted for Model 1 plus, ARIC test center, total energy consumption, diabetes, cigarette smoking, physical activity, income level and education.

Supplementary Table 2. Sample Characteristics. Baseline Characteristics of 15,428 women and men at baseline Visit 1 (1987-1989) in the Atherosclerosis Risk in Communities Study according to quintiles of **a) Low** Carbohydrate Animal Score or **b) Low Carbohydrate Plant Score** adjusted for age, and gender.

←**High Animal** Fat/Protein ←**Low Carb**ohydrate | **Lower Animal** Fat/Protein→ **Higher Carb**ohydrate→

Animal Score Quintile	Q1 n=3210	Q2 n=3180	Q3 n=3307	Q4 n=3112	Q5 n=2619	P-trend
**Carbohydrate,% of energy, mean±SD**	**38 ± 5**	**45 ± 5**	**49 ± 5**	**53 ± 6**	**61 ± 7**	
**Animal Protein (% of energy), mean±SD**	**18.4 ± 3.6**	**15.1 ± 2.8**	**13.2 ± 2.7**	**11.3 ± 2.4**	**8.5 ± 2.2**	
**Animal Fat (% of energy), mean±SD**	**27.9 ± 4.4**	**22.5 ± 3.1**	**19.4 ± 3.0**	**16.2 ± 2.9**	**12.3 ± 3.2**	
Age, mean±SD, y	53.9 ± 5.7	54.2 ± 5.8	54.3 ± 5.8	54.2 ± 5.8	54.3 ± 5.8	0.0126
Men, N(%)	1380 (43%)	1459 (46%)	1548 (47%)	1416 (46%)	1113 (42%)	0.7064
Black, N(%)	934 (29%)	856 (27%)	854 (26%)	802 (26%)	676 (26%)	0.0027
BMI (kg/m^2), mean±SE	28.5 ± 0.1	28.1 ± 0.1	27.6 ± 0.1	27.2 ± 0.1	27.0 ± 0.1	<0.0001
Diabetes, N(%)	532 (17%)	423 (13%)	362 (11%)	259 (8%)	234 (9%)	<0.0001
Hypertension, N(%)	1176 (37%)	1111 (35%)	1121 (34%)	1056 (34%)	905 (35%)	0.0151
Smoking, current, N(%)	863 (27%)	848 (27%)	874 (26%)	783 (25%)	650 (25%)	0.0404
Exercise activity, N(%) in quintile 5	554 (17%)	546 (17%)	590 (18%)	593 (19%)	495 (19%)	0.0195
College graduates, N(%)	902 (28%)	848 (27%)	877 (27%)	760 (24%)	564 (22%)	<0.0001
Household income >$50,000, N(%)	817 (26%)	773 (25%)	791 (24%)	711 (23%)	509 (20%)	<0.0001
Total energy intake (kcal), mean±SE	1546 ± 10	1621 ± 10	1668 ± 10	1634 ± 11	1662 ± 12	<0.0001
Plant Protein (% of energy), mean±SE	3.76 ± 0.02	4.22 ± 0.02	4.50 ± 0.02	4.77 ± 0.02	4.97 ± 0.02	<0.0001
Plant Fat (% of energy), mean±SE	10.8 ± 0.1	12.5 ± 0.1	13.2 ± 0.1	14.0 ± 0.1	14.1 ± 0.1	<0.0001
Saturated Fat (% of energy), mean±SE	14.6 ± 0.0	13.0 ± 0.0	11.9 ± 0.0	10.8 ± 0.0	9.1 ± 0.0	<0.0001
Monounsaturated Fat (% of energy), mean±SE	15.0 ± 0.0	13.5 ± 0.0	12.5 ± 0.0	11.6 ± 0.0	10.1 ± 0.0	<0.0001
Polyunsaturated Fat Fat (% of energy), mean±SE	5.1 ± 0.0	5.1 ± 0.0	5.0 ± 0.0	5.0 ± 0.0	4.7 ± 0.0	<0.0001
Dietary fiber (g), mean±SE	14.1 ± 0.1	16.4 ± 0.1	17.8 ± 0.1	18.5 ± 0.1	19.8 ± 0.2	<0.0001
Glycemic index	73.3 ± 0.1	74.0 ± 0.1	74.5 ± 0.1	75.0 ± 0.1	77.0 ± 0.1	<0.0001
Glycemic load	108.3 ± 1.1	133.9 ± 1.1	152.1 ± 1.0	163.0 ± 1.1	196.4 ± 1.2	<0.0001

Fruits, servings/d	1.52 ± 0.02	1.85 ± 0.02	2.01 ± 0.02	2.07 ± 0.02	2.35 ± 0.03	<0.0001
Vegetables, servings/d	2.90 ± 0.03	3.11 ± 0.03	3.17 ± 0.03	3.13 ± 0.03	3.17 ± 0.03	<0.0001
beef, pork, lamb as main dish, servings/d	0.44 ± 0.00	0.32 ± 0.00	0.26 ± 0.00	0.19 ± 0.00	0.12 ± 0.00	<0.0001
beef, pork, lamb as side dish, servings/d	0.34 ± 0.00	0.27 ± 0.00	0.23 ± 0.00	0.17 ± 0.00	0.12 ± 0.00	<0.0001
chicken with the skin, servings/d	0.19 ± 0.00	0.16 ± 0.00	0.14 ± 0.00	0.11 ± 0.00	0.09 ± 0.00	<0.0001
cheese, servings/d	0.48 ± 0.01	0.42 ± 0.01	0.39 ± 0.01	0.33 ± 0.01	0.26 ± 0.01	<0.0001
chicken without the skin, servings/d	0.56 ± 0.01	0.54 ± 0.01	0.50 ± 0.01	0.47 ± 0.01	0.40 ± 0.01	<0.0001
regular soft drinks, servings/d	0.17 ± 0.01	0.26 ± 0.01	0.36 ± 0.01	0.46 ± 0.01	0.82 ± 0.01	<0.0001

←**Higher Plant** Fat/Protein   Low Plant Fat/Protein→
←**Low Carb**ohydrate   Higher Carbohydrate→

Plant-Score Quintile	Q1 n=3555	Q2 n=3197	Q3 n=3254	Q4 n=2798	Q5 n=2624	P-trend
**Carbohydrate,% of energy, mean±SD**	**44 ± 7**	**47 ± 8**	**48 ± 9**	**49 ± 10**	**57 ± 9**	
**Plant Protein (% of energy), mean±SD**	**5.3 ± 1.1**	**4.7 ± 1.1**	**4.4 ± 1.1**	**3.9 ± 1.1**	**3.4 ± 0.8**	
**Plant Fat (% of energy), mean±SD**	**18.3 ± 4.4**	**14.5 ± 3.4**	**12.1 ± 3.3**	**9.7 ± 3.2**	**8.1 ± 2.7**	
Age, mean±SD, y	54.4 ± 5.7	54.1 ± 5.7	54.3 ± 5.8	54.2 ± 5.8	53.7 ± 5.8	<0.0001
Men, N(%)	1681 (47%)	1475 (46%)	1461 (45%)	1277 (46%)	1022 (39%)	<0.0001
Black, N(%)	600 (17%)	723 (23%)	861 (26%)	898 (32%)	1040 (40%)	<0.0001
BMI (kg/m^2), mean±SE	27.5 ± 0.1	27.5 ± 0.1	27.8 ± 0.1	27.7 ± 0.1	28.2 ± 0.1	<0.0001
Diabetes, N(%)	424 (12%)	350 (11%)	386 (12%)	332 (12%)	318 (12%)	0.2911
Hypertension, N(%)	1076 (30%)	1050 (33%)	1158 (36%)	1047 (38%)	1038 (40%)	<0.0001
Smoking, current, N(%)	944 (27%)	785 (25%)	837 (26%)	722 (26%)	730 (28%)	0.1966
Exercise activity, N(%) in quintile 5	610 (17%)	625 (20%)	599 (18%)	494 (18%)	450 (17%)	0.3663
College graduates, N(%)	986 (28%)	828 (26%)	833 (26%)	739 (26%)	565 (22%)	<0.0001
Household income >$50,000, N(%)	961 (28%)	825 (26%)	776 (24%)	604 (22%)	435 (17%)	<0.0001
Total energy intake (kcal), mean±SE	1612 ± 10	1638 ± 10	1622 ± 10	1582 ± 11	1677 ± 12	0.0486
Animal Protein (% of energy), mean±SE	12.8 ± 0.1	13.3 ± 0.1	14.0 ± 0.1	14.6 ± 0.1	13.0 ± 0.1	<0.0001
Animal Fat (% of energy), mean±SE	19.4 ± 0.1	19.8 ± 0.1	20.5 ± 0.1	21.3 ± 0.1	18.9 ± 0.1	<0.0001
Saturated Fat (% of energy), mean±SE	12.9 ± 0.0	12.3 ± 0.1	12.0 ± 0.1	11.8 ± 0.1	10.5 ± 0.1	<0.0001
Monounsaturated Fat (% of energy), mean±SE	14.7 ± 0.0	13.3 ± 0.0	12.5 ± 0.0	11.8 ± 0.0	10.1 ± 0.0	<0.0001
Polyunsaturated Fat Fat (% of energy), mean±SE	6.5 ± 0.0	5.4 ± 0.0	4.8 ± 0.0	4.3 ± 0.0	3.6 ± 0.0	<0.0001
Dietary fiber (g), mean±SE	18.3 ± 0.1	18.0 ± 0.1	17.2 ± 0.1	16.2 ± 0.2	16.0 ± 0.2	<0.0001
Glycemic index	75.1 ± 0.1	75.2 ± 0.1	74.6 ± 0.1	73.7 ± 0.1	74.7 ± 0.1	<0.0001
Glycemic load	134.6 ± 1.1	146.0 ± 1.1	146.7 ± 1.1	144.3 ± 1.2	179.7 ± 1.3	<0.0001
Fruits, servings/d	1.62 ± 0.02	1.84 ± 0.02	1.98 ± 0.02	2.10 ± 0.02	2.31 ± 0.03	<0.0001
Vegetables, servings/d	3.21 ± 0.03	3.28 ± 0.03	3.13 ± 0.03	2.99 ± 0.03	2.77 ± 0.03	<0.0001
nuts, servings/d	0.28 ± 0.00	0.14 ± 0.00	0.10 ± 0.00	0.07 ± 0.00	0.05 ± 0.01	<0.0001
peanut butter, servings/d	0.52 ± 0.01	0.27 ± 0.01	0.19 ± 0.01	0.13 ± 0.01	0.10 ± 0.01	<0.0001
dark or grain breads, servings/d	1.05 ± 0.02	0.89 ± 0.02	0.74 ± 0.02	0.64 ± 0.02	0.49 ± 0.02	<0.0001
chocolate, servings/d	0.34 ± 0.01	0.27 ± 0.01	0.22 ± 0.01	0.16 ± 0.01	0.13 ± 0.01	<0.0001
white breads, servings/d	0.85 ± 0.02	0.84 ± 0.02	0.79 ± 0.02	0.65 ± 0.02	0.63 ± 0.02	<0.0001
regular soft drinks, servings/d	0.15 ± 0.01	0.26 ± 0.01	0.33 ± 0.01	0.41 ± 0.01	0.98 ± 0.01	<0.0001
